# Gaze shifts during dual-tasking stair descent

**DOI:** 10.1007/s00221-016-4721-6

**Published:** 2016-07-11

**Authors:** Veronica Miyasike-daSilva, William E. McIlroy

**Affiliations:** 1 Center for Physical Ergonomics, Liberty Mutual Research Institute for Safety, 71 Frankland Road, Hopkinton, MA 01748 USA; 2 Department of Kinesiology, University of Waterloo, Waterloo, Canada; 3 Heart and Stroke Foundation Canadian Partnership for Stroke Recovery, Sunnybrook Health Sciences Centre, Toronto, Canada

**Keywords:** Stair locomotion, Vision, Gaze behaviour, Dual task

## Abstract

To investigate the role of vision in stair locomotion, young adults descended a seven-step staircase during unrestricted walking (CONTROL), and while performing a concurrent visual reaction time (RT) task displayed on a monitor. The monitor was located at either 3.5 m (HIGH) or 0.5 m (LOW) above ground level at the end of the stairway, which either restricted (HIGH) or facilitated (LOW) the view of the stairs in the lower field of view as participants walked downstairs. Downward gaze shifts (recorded with an eye tracker) and gait speed were significantly reduced in HIGH and LOW compared with CONTROL. Gaze and locomotor behaviour were not different between HIGH and LOW. However, inter-individual variability increased in HIGH, in which participants combined different response characteristics including slower walking, handrail use, downward gaze, and/or increasing RTs. The fastest RTs occurred in the midsteps (non-transition steps). While gait and visual task performance were not statistically different prior to the top and bottom transition steps, gaze behaviour and RT were more variable prior to transition steps in HIGH. This study demonstrated that, in the presence of a visual task, people do not look down as often when walking downstairs and require minimum adjustments provided that the view of the stairs is available in the lower field of view. The middle of the stairs seems to require less from executive function, whereas visual attention appears a requirement to detect the last transition via gaze shifts or peripheral vision.

## Introduction

Humans rely on vision to successfully interact with the surrounding environment. The role of vision in the control of movement is well documented for upper limb movements (Servos and Goodale 1994; Aglioti et al. 1995; Land et al. 1999) and for locomotion (Patla and Vickers 1997; Mohagheghi et al. 2004), with much of this work focussed on the role of foveal or central vision. Yet, for locomotor behaviours, the peripheral visual field may be particularly important given the relevant visual information available in a wide range of the field of view (Graci et al. 2010; Reed-Jones et al. 2014). However, in locomotor tasks requiring precise stepping, such as in stair navigation, the requirements for higher-resolution visual inputs have influenced research to focus on the role of foveal visual information for detection of stair physical properties (e.g. step rise and run). This emphasis on foveal vision may have led to an underestimation of the role of peripheral vision during locomotion. Additionally, peripheral vision appears to have an important role even for target behaviours during stair walking, such as grasping of handrails (King et al. 2010; Miyasike-daSilva et al. 2011). In the present study, we explored the role of foveal and lower field of view information during the task of navigating stairs.

Previous studies have revealed the importance of vision on stair climbing (Zietz and Hollands 2009; Miyasike-daSilva et al. 2011). Nevertheless, foveal fixations directed to stair features (e.g. steps, handrails) decrease when individuals are engaged in a concurrent visual task with little impact on locomotor behaviour (Miyasike-daSilva and McIlroy 2012). This decrease in foveal fixations suggests that peripheral vision is able to “capture” sufficient information about the spatial and physical properties of the stairs relative to the lower limbs required to guide stair walking. If so, a condition for using information from the peripheral visual field would be an optimal line of gaze to allow extraction of visual information from the extra-foveal visual field. The reliance on peripheral visual field information would provide additional advantages including minimizing the need to scan large field of view with foveal vision and releasing foveal vision to engage in other tasks.

In the current study, we sought to advance our fundamental understanding of the specific role of foveal vision during locomotion. We used a dual-task paradigm in which visual task conditions challenged the use of foveal vision and the associated peripheral vision to control stair locomotion. The specific point of gaze required to perform the visual task permitted measurement of timing and frequency of gaze shifts to probe the dependence on lower peripheral visual information to control stair walking. For instance, a line of gaze directed forward could limit the view of the stair in the peripheral field of view during stair descent. In this way, we imposed a “natural” task challenge that restricts the available foveal and peripheral visual information for stair locomotion.

Dual-task walking has well-known effects on both gait and cognitive performance, and such effects have also been revealed during stair navigation. Previous literature has shown that dual tasking is associated with changes in gait speed, lower limb kinematics and kinetics, and foot clearance during stair navigation (Qu and Hu 2014; Telonio et al. 2014; Madehkhaksar and Egges 2016). In other studies, it has been also observed that the performance in the concurrent cognitive task decreases during stair walking (Ojha et al. 2009; Miyasike-daSilva and McIlroy 2012). Nevertheless, others have found no clear decrement in cognitive performance associated to stair walking (Madehkhaksar and Egges 2016), raising the importance of prioritization between concurrent tasks modulated by either specific instructions during the experiment, or individual’s personal choice. In the present study, we explored dual-task trade-offs and inter-individual differences in combining adaptive strategies when facing the restriction in the lower field of view while dual tasking. Yet, we aimed to demonstrate the presence or absence of adaptive behaviours that would reveal a potential role of foveal/peripheral vision during stair walking.

In the present study, a visual task was presented on a monitor located at different heights in order to restrict (monitor at a high position) or facilitate (monitor at a low position) the view of the stairs in the lower field of view. We hypothesized that information from the lower peripheral field of view is specifically important in stair transition phases (ground-to-stairs and stairs-to-ground). We anticipated that restriction in the lower field of view would affect gaze behaviour, stair walking time, and reaction time latency/accuracy in the visual task, while navigating transitions. For gaze behaviour, we hypothesized that individuals would execute discrete transient downward gaze shifts within one stride prior to transition steps when the use of the lower peripheral visual field was limited. For locomotor behaviour, we hypothesized that the natural restriction of the lower field of view would increase the total time to walk downstairs and the single support duration in the transition step. For reaction time performance, we hypothesized increased latency and reduced accuracy when the view of the stairs in the lower field of view was limited highlighting the additional attentional/cognitive burden with limited peripheral visual information guiding locomotion.

## Methods

### Participants

 Ten healthy young adults (five females, 23.8 ± 3.0 years, 168 ± 8 cm height) with normal vision or vision corrected-to-normal (with contact lenses), with binocular visual acuity of 20/20 or higher (Snellen test), and with mean contrast sensitivity of 1.79 ± 0.05 log (Mars Letters) participated in this study. Participants self-reported to be free of motor or neurological condition affecting their balance or ability to traverse stairs. Informed consent was obtained from all individual participants, and all procedures performed in the study were in accordance with the ethical standards of the institutional research committee and with the 1964 Helsinki Declaration and its later amendments or comparable ethical standards.

### Protocol

Participants descended a seven-step staircase (96.5 cm wide, 18 cm rise, and 25.5 cm tread). Handrails were present on each side of the stairs. Participants wore a safety harness attached to a retractable lanyard running along a cable above the stairs. Before each trial, the view of the stairs and the handrails was occluded with a piece of cardboard held in front of the participant by an assistant. The participant’s start position varied across three different distances from the edge of the top step (1.2, 1.4, or 1.6 m) to prevent memorization of distance from the stairs. When the trial started, the cardboard was removed and the participant descended the stairs at their natural pace and continued walking on ground level for 3–4 steps. At the end of each trial, participants were asked to return to the start position upstairs.

The visual reaction time task consisted of responding to a visual stimulus displayed on a LCD monitor. The letters “X” and “O” were randomly presented on the monitor at a proportion of occurrence of 3/1, respectively. Stimuli were presented for 100 ms at random intervals (750–1250 ms). Participants were asked to click on a wireless mouse button every time they saw an “X”. Participants were provided with practice trials in the visual task at beginning of the data collection session.

**Fig. 1:**
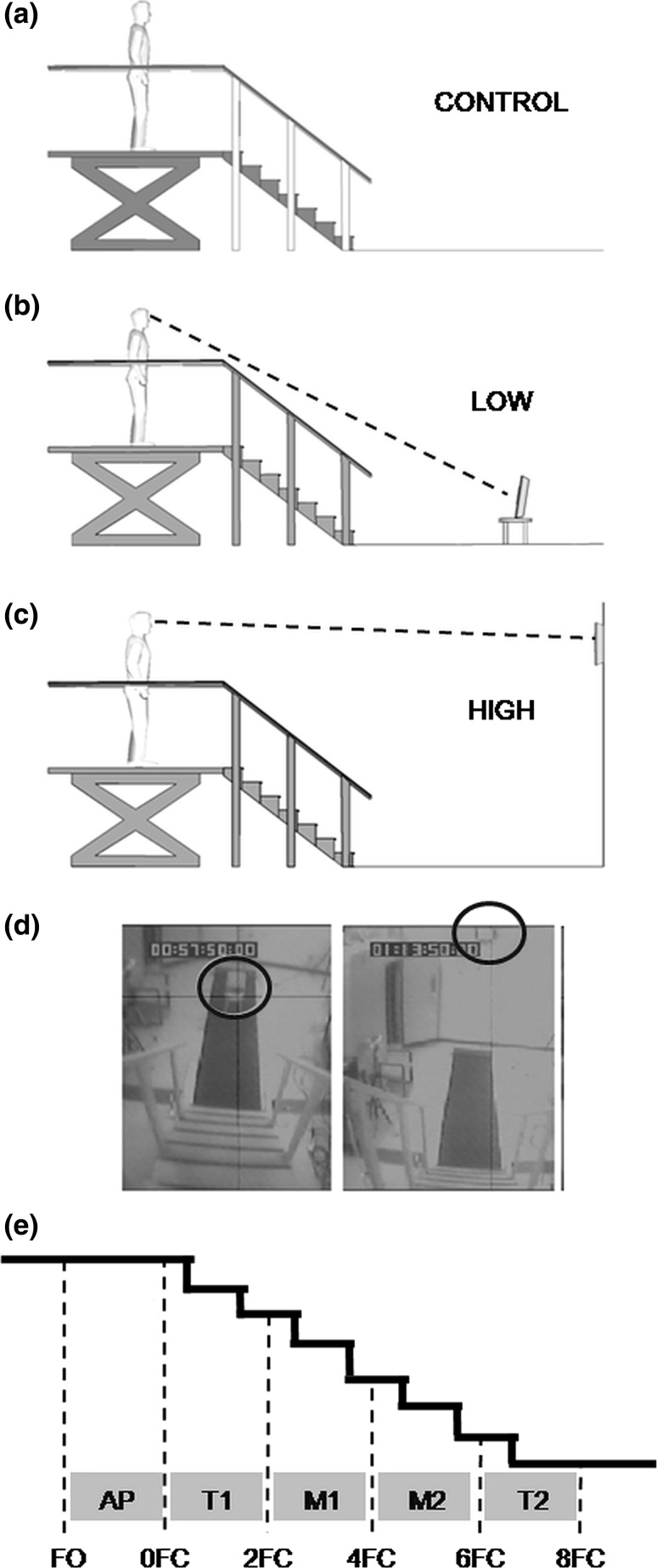
Experimental protocol. **a** Unrestricted walking (CONTROL); **b** dual task with peripheral vision facilitation (LOW); **c** dual task with peripheral vision restricted (HIGH); **d** video frames captured from head-mounted camera, with the participant at the *top* of the stairs; the images show the monitor (*circle*) and steps during HIGH (*left*) and LOW (*right*) conditions; *dashed line* in **a** and **b** illustrates participant’s line of gaze oriented towards the monitor; **e** classification scheme for participants’ step location when descending the stairs. AP, approach; T1, first transition; M1, first midstep region; M2, second midstep region; T2, second transition; −2FC, two foot contacts before stepping on the stair; 0FC, last foot contact before the stair; 2FC, foot contact on the step 2; 4FC, foot contact on the step 4; 6FC, foot contact on the step 6; 8FC, first foot contact out of the stairs

Stair descent was performed in three conditions (Fig. 1): (a) *CONTROL*: stair walking alone (Fig. 1a), with no specific instruction to participants on where they should look; (b) LOW: stair walking and visual task performed concurrently, with the monitor located downstairs 50 cm above the ground level (Fig. 1b), which corresponded to a visual angle of 24°–27° below the horizontal gaze line when participants were standing on the top of the stairs; (c) HIGH: stair walking and the visual task performed concurrently, with the monitor mounted on the wall, 3.5 m above the ground level, approximately at participants’ eye height when standing upstairs (Fig. 1c). By changing the monitor location, the view of the stairs in the lower field of view was facilitated (LOW) or restricted (HIGH; Fig. 1d). The monitor location in HIGH restricted participants from seeing the steps in the lower peripheral field of view as they walked downstairs, since it required participants to direct their gaze upwards to look at the monitor to perform the visual task. In LOW and HIGH conditions, participants performed the visual task continuously throughout the entire stair walk. No specific instruction was given to participants on which task they should prioritize. In CONTROL, the monitor was removed from the walkway and wall. Participants carried the wireless mouse on their preferred self-selected hand (right hand for all participants). No specific instruction was given with regard to use of handrails. Each participant performed a total of 42 trials split into two even blocks. Each block had five trials for each condition (CONTROL, HIGH, and LOW). The conditions were presented in random order within each block. In each block, three additional trials with the visual task performed alone while standing upstairs for 10 s were randomly included within each HIGH and LOW conditions.

### Data acquisition and analysis

 Footswitches (B&L Engineering, Tustin, CA, USA) inside of participant’s shoes provided temporal measurement of their steps. Footswitch data were collected at 240 Hz using a customized LabVIEW program (National Instruments, Austin, TX, USA). Participants’ stepping location was classified in one of the following step regions (Fig. 1e): (a) *approach (AP)*, the first foot off (FO) to the last foot contact prior to the stairs (0FC); (b) *first transition* (T1), from 0FC to the foot contact on step 2 (2FC); (c) *first midsteps* (M1), from 2FC to the foot contact on step 4 (4FC); (d) *second midsteps* (*M2*), from 4FC to the foot contact on the step 6 (6FC); (e) and *second transition* (*T2*), from 6FC to the first foot contact on ground level (8FC). *Walk time for each stair region* (AP, T1, M1, M2, and T2) and *single support duration* in each step (from foot off to foot contact) were calculated. A video camera recorded participants walking on the stairs, which was used to code handrail use frequency.

Eye movements were recorded with a head-mounted eye tracker 5000 (ASL, Bedford, MA, USA) at a rate of 60 Hz. The eye tracker was calibrated with the nine-point calibration method with 1° accuracy over the stair area. The eye tracker system provided video outputs of the eyeball, and the gaze location superimposed on the participant’s field of view (scene view). Video recordings were analysed frame by frame to identify gaze shifts downward. A gaze shift downward was determined directly from the video recordings when a clear downward movement of the eye was detected resulting in a line of gaze away from the monitor displaying the visual task. The specific gaze location following a gaze shift was not determined because, in most cases, final gaze location was beyond the scene view limits of the eye tracker system. The *time looking down* was calculated as a percentage of trial time. *Gaze shift frequency* was calculated as the percentage of trials with downward gaze shifts according to participant’s location on the stairs (AP, T1, MS1, MS2, T2, as determined from footswitch data) when a gaze shift occurred.

For the visual task, the time for each stimulus delivery and button press was recorded via a custom LabVIEW program. Visual task performance was assessed by reaction time and accuracy. *Reaction time* was calculated from the appearance of the stimulus on the screen to the button press. *Accuracy* was calculated as the percentage of correct responses. A response was considered correct when participants responded to the “X” stimulus, or did not respond to the “O”. Mean reaction time and accuracy were calculated for each step region per condition (only responses in which both stimulus and response occurred within the same step region were included). The single-task performance of the visual task was computed by the mean values from the six standing trials for each HIGH and LOW conditions. Dual-task cost was calculated as the percentage of change in the mean dual-task (DT) performance relative to single-task (ST) performance: ${\text{DT}}_{\text{cost}} = \left( {{\text{DT}} - {\text{ST}}} \right)/{\text{ST}} \times 100$. Therefore, negative values in dual-task cost indicate a decrease in accuracy or shorter reaction times in dual-task condition compared with single task.

The time looking down was analysed by a one-way repeated measures ANOVA with task condition as a factor with three levels (CONTROL, LOW, and HIGH). Gaze shift frequency, walk time, reaction time, and accuracy were all analysed using a two-way repeated measures ANOVA with task condition and stair region as the two factors. Single support duration was analysed using a two-way ANOVA with condition and specific step number (from −1 to 7) as factors. Post hoc analysis (Tukey’s adjustment) was used to characterize the differences across conditions and step regions with significance level set at 0.05. Individual strategies were assessed by qualitatively analysing combination of adaptive strategies across participants.

## Results

### Gaze behaviour

**Fig. 2:**
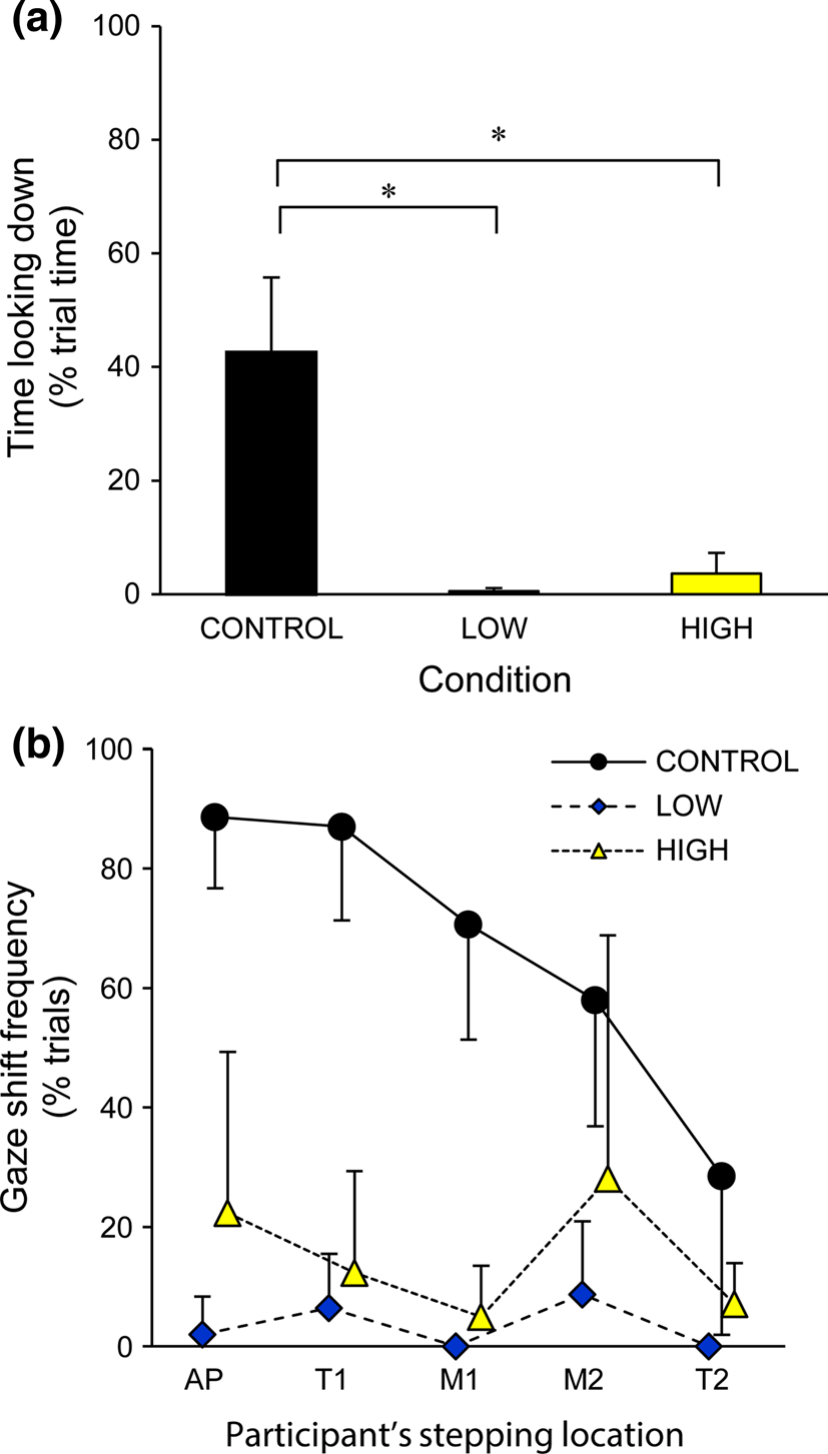
**a** Mean time looking down across each condition. **b** Gaze shift frequency across conditions according to participants’ stepping location on the stairs. *p < 0.0001

Overall, downward gazes were reduced in both LOW and HIGH compared with CONTROL. Specifically, compared with CONTROL, in LOW and HIGH, downward gazes were shorter in duration (421 ± 50, 250 ± 62, and 274 ± 96 ms, respectively) and less frequent (60.9 ± 15.6, 1.9 ± 1.9, and 11.8 ± 12 % of trials). Additionally, downward gaze shifts were not observed in three and one participants in LOW and HIGH, respectively, while all ten participants performed downward gaze shifts in CONTROL. This reduction in downward gaze behaviour is evidenced by a significant main effect of experimental conditions on the total time looking down [*F*(2,18) = 87.88, *p* < 0.0001; Fig. 2a].

For gaze shift frequency, there was a main effect for condition [*F*(2,18) = 98.73, *p* < 0.0001], step region [*F*(4,36) = 13.18, *p* < 0.0001], and an interaction between condition and step region [*F*(8,72) = 8.75, *p* < 0.0001, Fig. 2b]. Overall, gaze shift frequency was decreased in LOW and HIGH compared with CONTROL in all step regions. In CONTROL, gaze shift frequency decreased as participants walked downstairs. In LOW and HIGH, there was an overall reduction in gaze shift frequency across all step regions. Within HIGH condition, although the observed increase in downward gaze shift frequency in step regions prior to transitions (AP and M2) did not reach statistical significance when compared with other step regions, there was a large standard deviation (22.33 ± 26.94 and 28.11 ± 40.72 %, for AP and M2, respectively).

### Locomotor behaviour

**Fig. 3:**
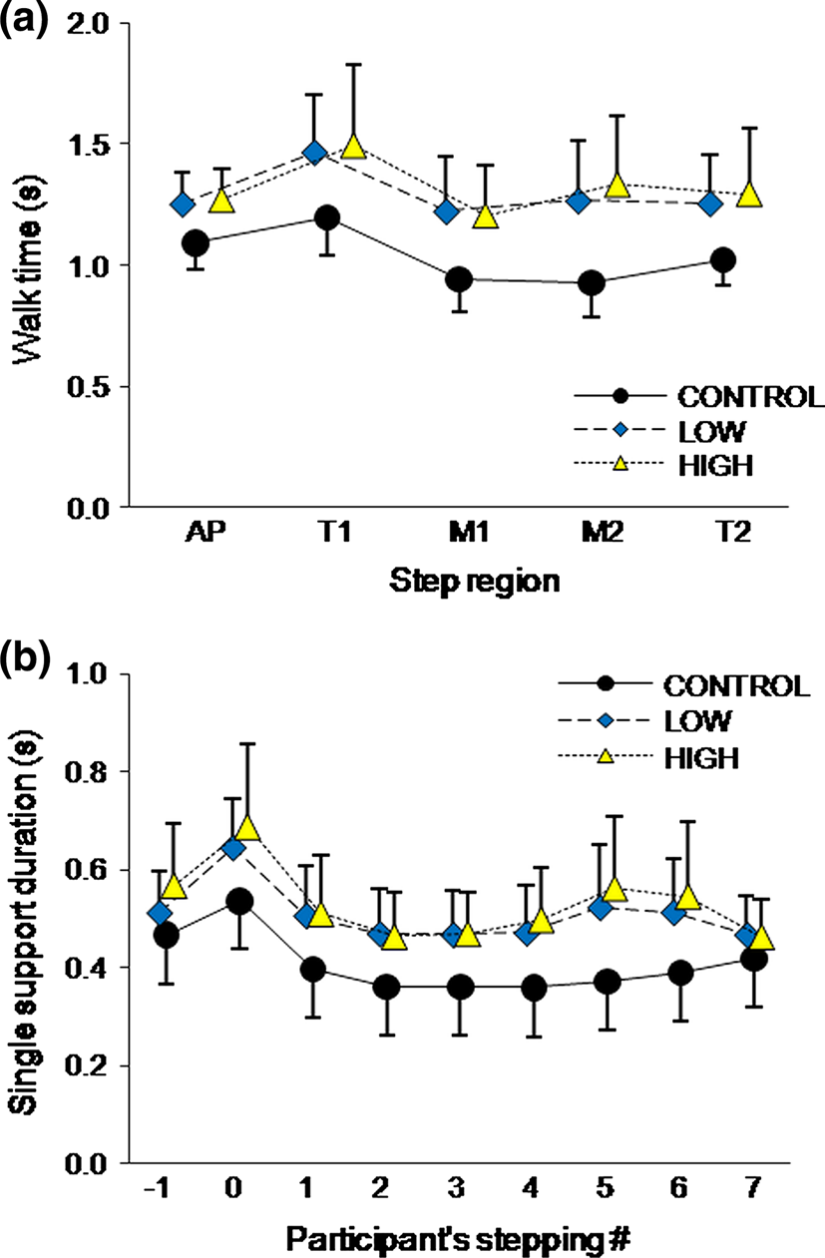
**a** Walk time (s) across each experimental condition and step region. T1, transition 1; M1, midsteps 1; M2, midsteps 2; T2, transition 2. **b** Single support duration in each step by condition

For walk time, there was a main effect of task condition [*F*(2,18) = 19.83, *p* < 0.0001], step region [*F*(4,36) = 11.14, *p* < 0.0001], and a condition by step region interaction [*F*(8,72) = 4.2, *p* = 0.0004; Fig. 3a]. Walk time was increased in LOW and HIGH compared with CONTROL in all step regions excluding the first transition. There was no statistical difference between LOW and HIGH in walk time.

Video recordings suggested that some participants seemed to delay foot contact near the transitions as an attempt to search for the transition step during the dual-task trials. In order to confirm this observation, the single support duration in each step was analysed (Fig. 3b) and it was evidenced by a main effect of condition [*F*(2,18) = 17.36, *p* < 0.0001], step [*F*(6,72) = 22.47, *p* < 0.0001], and an interaction between task condition and step location [*F*(16,144) = 5.69, *p* < 0.0001]. Single support duration was longer in every step in both LOW and HIGH compared with CONTROL (with exception of step −1 in LOW and step 7 in LOW and HIGH). Single support duration was also similar between LOW and HIGH (except for step −1). Although the single support duration in the transitions was not statistically longer than in other steps, there was a larger standard deviation in steps “0”, “5”, and “6” during HIGH, indicating increased variability in single support duration between participants.

Four of the ten participants used the handrail at some point in the study. Only one participant held the handrail during CONTROL condition, and this occurred in only one trial. In LOW, handrail was used in 26 % of all trials (three participants), while in HIGH, handrail was used in 34.7 % of the trials (four participants).

### Visual task performance

As expected, dual-task significantly increased reaction time compared with single-task in LOW [*F*(1,9) = 18.34, *p* = 0.002] and HIGH [*F*(1,9) = 32.51, *p* = 0.0003]. Similarly, accuracy was significantly reduced during dual task compared with single task in LOW [*F*(1,9) = 12.42, *p* = 0.0065] and HIGH [*F*(1,9) = 33.39, *p* = 0.0003].

For reaction time, there was a main effect of visual task condition [*F*(1,9) = 5.65, *p* = 0.041] and step region [*F*(4,36) = 7.57, *p* = 0.0002; Table 1]. Specifically, reaction time was increased in HIGH compared with LOW. Additionally, reaction time was faster at the first midstep (M1) compared with all other step regions. For accuracy, there was no statistically significant differences between the HIGH and LOW visual tasks (*p* > 0.05), step regions (*p* = 0.090), and there was no significant interaction between visual task and step region (*p* = 0.072).

**Table 1:** Means ± SD for reaction time (ms) and accuracy (%correct responses) during single- and dual-task conditions for HIGH and LOW, and across step regions

	LOW		HIGH		Overall mean^b^	
	Reaction time (ms)	Accuracy (%)	Reaction time (ms)	Accuracy (%)	Reaction time (ms)	Accuracy (%)
Single task^a^	314 ± 25	91.0 ± 5.3	318 ± 24	89.4 ± 8.9		
Dual task^a^	337 ± 45*	82.7 ± 15.1	351 ± 48*	80.2 ± 15.2		
AP	363 ± 42	82.8 ± 5.7	375 ± 48	78.2 ± 10.0	369 ± 44	80.5 ± 8.27
T1	334 ± 42	80.5 ± 10.5	352 ± 52	80.7 ± 13.6	343 ± 37	80.6 ± 11.9
M1	302 ± 28	84.3 ± 16.7	305 ± 41	91.5 ± 10.4	303 ± 34**	87.9 ± 14.0
M2	348 ± 43	75.8 ± 24.2	362 ± 39	71.1 ± 21.3	355 ± 40	73.4 ± 22.3
T2	339 ± 50	90.0 ± 10.4	361 ± 56	79.7 ± 16.2	350 ± 53	84.8 ± 14.3

* Statistical difference between LOW and HIGH (*p* < 0.05)

** Statistical difference between M1 and other step regions (*p* < 0.0001)

^a^Means across all step regions

^b^Means across conditions for each step region (approach, T1, M1, M2, T2)

### Individual strategies

Inspection of individual data revealed that four participants drastically reduced walking speed during the most restricted condition (HIGH), while the other six participants showed only a small reduction in walking speed compared with CONTROL. We further investigated individual strategies by dividing participants in two subgroups (Table 2). Participants were assigned in the “slow walker” subgroup if they increased walk time over 30 % in HIGH and LOW compared with CONTROL. Handrail was used more often for slow walkers (*N* = 3) than for fast walkers (*N* = 1). Downward gaze shifts were more often performed by fast walkers than by slower walkers. In the visual task, three slow walkers showed the lowest dual-task cost in reaction time, while one participant in this group showed higher dual-task cost levels similar to the fast walkers. All slow walkers showed lower dual-task cost in accuracy in LOW; however, they appear similar to fast walkers in HIGH.

**Table 2:** Summary of individual data for locomotor behaviour, gaze behaviour, secondary task performance, and handrail use

Participant	Locomotion					Gaze			Visual task										Handrail use		
	Walk time (s)			%Change		Gaze shifts (% trials)			Reaction time (ms)			Reaction time cost (%)^a^		Accuracy (%)			Accuracy cost (%)^a^		# trials/total # trials		
	CNT	LOW	HIGH	LOW	HIGH	CNT	LOW	HIGH	Single task	LOW	HIGH	LOW	HIGH	Single task	LOW	HIGH	LOW	HIGH	CNT	LOW	HIGH
*Fast walkers <30* % *walk time increase*																					
P1	4.3 ± 0.6	4.5 ± 0.2	4.4 ± 0.2	3.6	2.0	100	0	10	295 ± 29	324 ± 46	360 ± 46	9.9	22.2	90.6 ± 9.9	90.2 ± 12.0	82.1 ± 14.9	−0.4	−9.4	0/10	0/10	0/10
P2	4.7 ± 0.2	5.1 ± 0.3	5.0 ± 0.3	8.5	6.0	100	30	100	312 ± 22	355 ± 41	342 ± 57	13.9	9.8	93.9 ± 12.6	81.1 ± 15.0	79.4 ± 13.8	−13.6	−17.8	0/10	0/10	0/10
P3	4.6 ± 0.3	5.1 ± 0.2	5.2 ± 0.2	12.2	13.4	100	22	60	312 ± 14	362 ± 43	359 ± 30	15.8	15.1	97.1 ± 4.1	89.2 ± 11.2	95.3 ± 10.0	−8.1	−2.0	0/9	0/9	0/10
P4	4.8 ± 0.2	5.7 ± 0.1	5.4 ± 0.2	18.8	13.8	100	50	100	320 ± 37	351 ± 44	356 ± 63	9.5	11.0	96.2 ± 5.8	80.0 ± 11.9	80.3 ± 6.8	−16.8	−19.9	0/7	0/8	0/10
P5	5.0 ± 0.2	5.6 ± 0.3	5.7 ± 0.1	12.2	14.4	100	0	10	285 ± 20	309 ± 25	348 ± 57	8.2	22.1	94.5 ± 5.1	73.0 ± 16.1	84.2 ± 15.2	−22.8	−14.1	0/9	0/9	0/10
P6	5.9 ± 0.1	6.9 ± 0.3	7.2 ± 0.6	16.9	21.5	100	30	100	331 ± 28	383 ± 59	384 ± 36	15.4	15.8	91.2 ± 8.6	80.3 ± 13.2	80.8 ± 14.0	−11.9	−13.0	0/8	7/10	7/10
*Slow walkers >30* % *walk time increase*																					
P7	4.8 ± 0.5	6.2 ± 0.1	6.6 ± 0.6	31.5	37.9	100	10	67	326 ± 22	333 ± 46	338 ± 40	2.2	3.7	84.9 ± 12.5	86.5 ± 8.5	72.2 ± 14.5	1.8	−14.7	0/10	0/10	0/9
P8	3.8 ± 0.3	5.6 ± 0.2	6.3 ± 0.2	46.4	63.3	100	0	20	373 ± 28	377 ± 43	386 ± 27	1.2	3.5	89.4 ± 9.4	89.4 ± 10.2	68.6 ± 19.9	0.0	−23.3	1/10	0/10	10/10
P9	4.7 ± 0.7	6.4 ± 0.5	7.7 ± 0.6	37.4	63.8	100	10	0	305 ± 19	349 ± 30	379 ± 46	14.7	24.3	94.9 ± 6.2	90.7 ± 10.0	85.0 ± 7.3	−4.4	−10.9	0/10	10/10	10/10
P10	4.7 ± 0.3	7.9 ± 0.6	8.1 ± 1.6	65.8	69.9	100	10	22	300 ± 19	308 ± 29	301 ± 38	2.6	0.3	92.1 ± 9.2	90.2 ± 11.7	77.2 ± 14.6	−2.1	−16.6	0/10	8/10	7/9

^a^Dual task cost was calculated as the percentage of change in performance in the dual task (DT) condition compared to single task (ST) performance as: ${\text{DT}}_{\text{cost}} = \left( {{\text{DT}} - {\text{ST}}} \right)/{\text{DT}} \times 100$; negative numbers in dual-task cost represent decrease in accuracy in dual tasking compared to single task

**Fig. 4:**
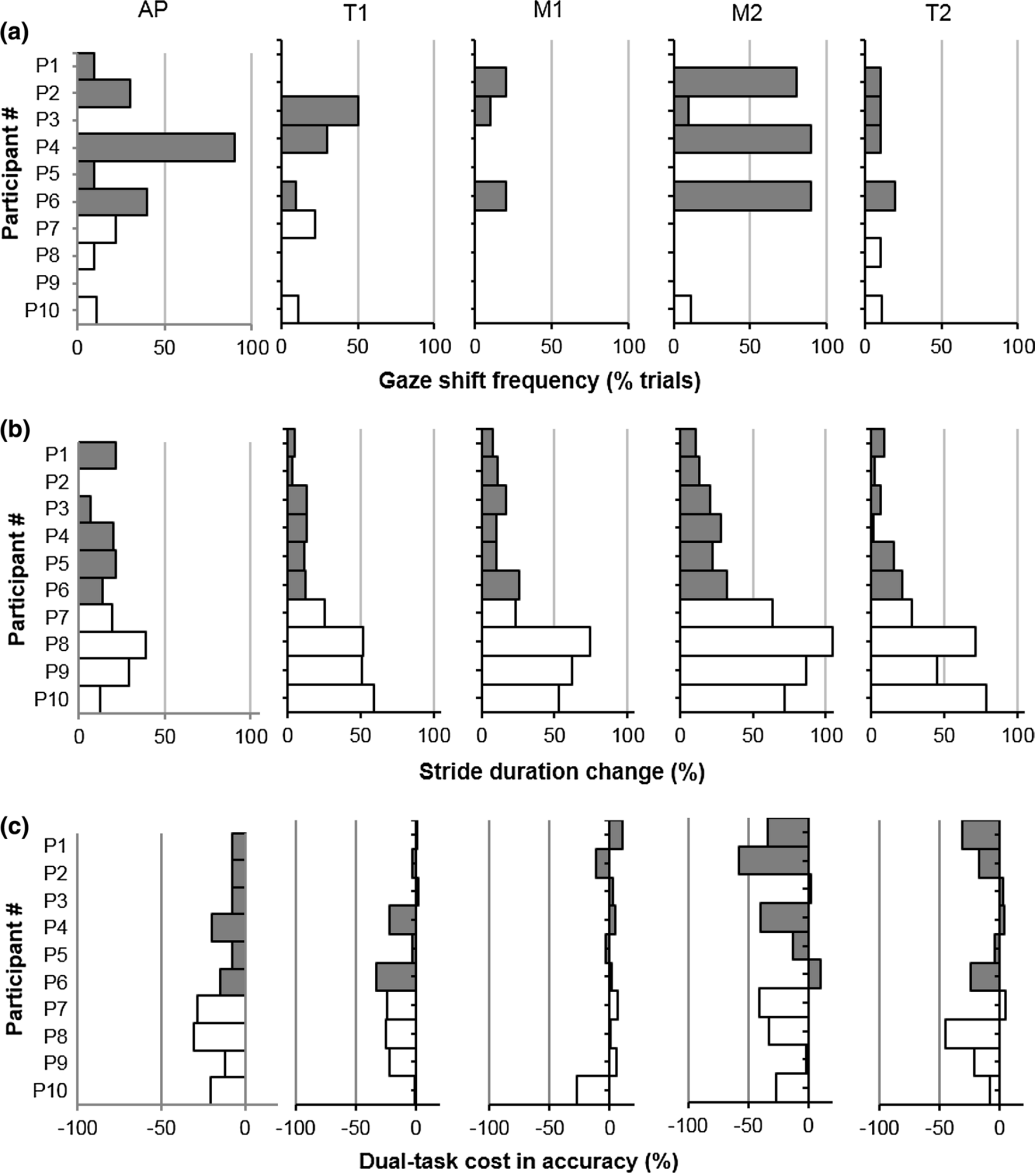
Summary of individual strategies employed by slow and faster walkers. **a** Frequency of trials with gaze shifts in each step region during the HIGH condition for each subject; “fast walkers” in *grey bars* and “slow walkers” in *white bars*. **b** Mean walk time in each step region for fast and slow walkers. **c** Dual-task cost in accuracy for the secondary task in each step region in the HIGH task condition

The classification as “slow walkers” and “fast walkers” accounted for the variability found in the group analysis in specific stair regions. Figure 4a–c shows individual data combining gaze, locomotion, and visual task variables in HIGH condition. The large gaze shift variability prior to transitions in HIGH resulted from the variability within the fast walker group (Fig. 4a). For instance, in M2, half of the fast walkers performed gaze shifts in most of the trials (*N* = 3), one participant looked down for only one trial (P3) and the remaining two participants did not perform gaze shifts in any trial. Large variation in gaze behaviour could also be observed in the approach phase. Slow walkers showed very low frequency or absence of gaze shifts throughout all step regions. Figure 4b shows the percentage of change in stride duration in HIGH relative to CONTROL (positive values indicate increased stride duration HIGH). Overall, fast walkers were less likely to slow down throughout all step regions, while slow walkers slowed down in all step regions. This individual difference in stride duration contributed for the large standard deviation in stride time observed in Fig. 3b. Finally, Fig. 4c shows individual data for dual-task cost on accuracy in the visual task in each step region comparing slow and fast walkers in the HIGH condition. In M2, dual-task cost largely varied between participants, which accounted for the large standard deviation in accuracy in this step region compared with the other steps as observed in Table 1.

## Discussion

The aim of this study was to explore the role of central vision and lower visual field during stair descent. By presenting a visual task in two different locations, the line of gaze was naturally influenced, which facilitated or limited the view of the stairs in the lower field of view during stair descent. In the presence of a concurrent visual task, downward gaze shifts were reduced compared with an unrestricted condition, indicating that continuous foveal feature extraction is not a requirement for stair walking. Gaze shift frequency remained low independently if the visual task facilitated or restricted the use of the lower visual field to extract visual information about the stairs. Previous studies showed long periods with the line of gaze directed to the walking surface (Patla and Vickers 1997; Marigold and Patla 2007; Miyasike-daSilva et al. 2011), which was related to the need to acquire visual information to guide locomotion. Recently, it was suggested that this line of gaze simply represents natural gaze tendencies due to task requirements and that gaze fixations could actually be reduced with minimal effects on gait performance during stair ascent (Miyasike-daSilva and McIlroy 2012). The current study also provides evidence against the need for continuous gaze directed to the walking surface during stair descent.

Considering that the human lower field of view extends for more than 60° inferiorly from the midline (Millodot 2014), the view of the steps (via peripheral vision) could have been facilitated when the line of gaze was directed to the monitor located downstairs, and consequently minimized the need for additional downward looks in the middle of the stairs. Studies have shown that such peripheral visual information is appropriate to implement changes in gait (Marigold and Patla 2007; Timmis et al. 2009; Graci et al. 2010). When the monitor was elevated, however, the large variability in gaze shifts found in the steps preceding the transition to ground level suggests that at least some participants looked down as an attempt to regain the view of the steps within the lower visual field, similar to “the last look” referred to by Rosenbaum (2009). For those participants who rarely looked down, two possible explanations could be explored. First, it is possible that participants were still able to infer information regarding stair features, since a specific restriction in the peripheral visual field was not applied in the current study (e.g. occluding the lower visual field). For instance, the view of the handrails could provide information regarding the general location of the transition steps. In fact, some participants reported that the view of the handrails (in the peripheral field of view) served as a “cue” for the beginning and the end of the stairs. Second, taking into account the size of the human visual field, the view of the first steps may be available in the field of view while fixating on the monitor mounted on the wall; however, in the last few steps, individuals may have used a stored representation of the stairs, considering that locomotion can be guided by memory of the environmental layout within short periods of time (Thomson 1983). Future studies investigating the range of peripheral vision for stair walking in controlled conditions will provide more substantial information of this role. Nevertheless, the present study demonstrated a reduction in downward gaze shifts during dual-tasking stair descent. This reduction suggests that gaze fixations can be minimized during stair walking, and supports the notion that the lower peripheral vision provides relevant visual information to control stair walking.

Individuals varied their behaviour when the visual task restricted the view of the stairs. To deal with the dual-task conditions, individuals adopted different strategies such as walking slower, using handrails, and/or looking down. Arguably this could suggest the need for continuous use of foveal vision to guide stair walking. Alternatively, rather than a visual requirement, the inherent cognitive load from the dual task could have been the factor contributing to the adoption of a more cautious gait strategy. Previous research has shown an increased attentional cost during stair walking (Ojha et al. 2009; Telonio et al. 2014), which was likely the case in the current study. It has been demonstrated that the performance of a central visual task diminishes the ability to detect stimuli presented in the peripheral visual field, creating a reduction in the functional field of view, which is the total visual field area in which a stimulus can be detected (Ball et al. 1988; Brabyn et al. 2001). The reduction in the functional field of view with increased cognitive load was observed in a static visual field test (Williams 1982) and under increased postural challenge (Reed-Jones et al. 2014). Therefore, the reduction in the functional field of view may also happen at the same or to an even greater degree during locomotion given the challenges to maintain balance control. In the present study, the narrowing in the functional visual field could have limited the use of visual information from the lower field of view resulting in more cautious locomotor strategies. Reduction in gait speed is common during dual tasking in more challenging locomotor conditions, such as obstacle avoidance, which is thought to be related to the increased executive requirements under more complex conditions (Siu et al. 2008). Similar effect was previously shown during stair ascent, where decrements in walking performance were linked to executive load rather than visual demands (Miyasike-daSilva and McIlroy 2012). Therefore, the results from the current study suggest that the changes observed were due to the restriction of the useful field of view linked to the cognitive demands of the dual task.

The performance in the visual task in the current study appeared to support this notion of useful field of view. Reaction time was longer during stair walking, which was probably an effect of prioritizing locomotion and balance control over the visual task, a common strategy adopted by healthy individuals while dual-tasking (Yogev-Seligmann et al. 2008). When the visual task restricted the view of the stairs (HIGH), even longer reaction times were observed. Head orientation was not directly measured in this study; however, it would be assumed that the HIGH condition would cause varying degrees of neck extension, while the LOW condition would cause some degree of neck flexion. Consequently, the reaction time responses may have been influenced by activity in the neck muscles and/or vestibular inputs due to the changes in head orientation. For instance, neck flexion in the LOW condition could have contributed to reduced reaction time responses as previously demonstrated with saccadic reaction time responses (Fujiwara et al. 2000). However, given that the differences in the reaction time responses between LOW and HIGH single tasks were not significant (Table 1), we believe that the influence of head orientation was modest in the current study. The potential impact of altered head position (and therefore neck muscle activity, proprioception, and vestibular inputs) should continue to be considered in future studies exploring gaze changes that are associated with head movements. Alternatively, the increased reaction times in HIGH dual task could be related to managing the requirements for functional field of view. The narrowing in the functional visual field is well established in visual assessment and shows a relationship with driving skills and ageing (Ball et al. 1988; Brabyn et al. 2001; Rogé et al. 2005). However, the relationship is less clear between functional visual field and activities requiring balance control, such as during locomotion. It could be possible that the reduction in the functional field of view was minimized for the purpose of maintaining control of locomotion, which in turn resulted in decreased performance of the central visual task as more executive resources where allocated to monitor the peripheral field of view. Future studies associating the concept of functional visual field and tasks challenging balance control will be able to explore the limits of the peripheral visual information in more complex contexts.

One study design factor that may have influenced the current findings was the need to carry the wireless mouse in the preferred hand. Handrail use increased during dual tasking even though carrying a wireless mouse in one hand may have reduced the opportunity for handrail use. However, in the current study, less than half of participants used the handrail, which is slightly above the general handrail use frequency in the young population of approximately one-third of stair users (Cohen and Cohen 2001). While current results may indicate that even in more challenging situations such as dual-tasking and carrying objects, holding a handrail is still not a predominant strategy by young adults this may be a more important matter in those more dependent on upper limb support (e.g. older adults).

In the current study, dual tasking influenced walking time similarly during transitions and midsteps, suggesting that transitions and midsteps have similar requirements in terms of executive load, and consequently, similar strategies could be used to compensate for the dual-task demand. It should be considered, however, that participants were likely able to see the first step in the lower visual field when approaching the stairs, even when looking at the monitor mounted high on the wall, which may have reduced the uncertainty regarding the beginning of the stairs and the need for major changes in gait speed. Additionally, participants widely varied in the degree they reduced gait speed prior to the second transition, which may have contributed to the lack of effect of transitions on walk time. Since participants did not receive specific instruction on which task they should prioritize, this likely leads to variations in the strategies selected and affected the ability to detect any statistically different changes in gait speed in transitions. Similarly, although the phases prior to transition steps did not affect significantly reaction times and accuracy, it is interesting to note that accuracy widely varied prior to the last transition. This could mean that at least some individuals shifted their attention to gait at the end of the stairs, as an attempt to detect the end of the stairs. The need to switch attentional resources to gait might be associated with increased downward gaze shifts performed by a few participants (e.g. foveal gaze transiently diverted from the stimulus). In the case of participants who did not perform gaze shifts, we speculate that a switch of attentional resources to the peripheral visual field without an associated gaze shift could have occurred.

The shortest reaction times occurred during walking in the midsteps, which is in agreement with previous study showing that dual-task cost is reduced during steady-state stair descent (Telonio et al. 2014). This finding may suggest a reduction in executive requirements to control gait in the middle steps by using knowledge gained from the interaction with the first steps, reliance on somatosensory information and/or a stored representation of the stairs. It was previously observed that the distance in which the foot clears the steps reduces along successive steps on a staircase, which is associated with an accommodation of foot trajectory to the steps dimension, assuming that the steps are similar within a staircase (Hamel et al. 2005). With this highly predictable step-to-step geometry, a greater reliance on stored representation of the expected step configuration and reduction in visual attentional demands in the midsteps most likely contributed to improvement in the visual task observed in the current study.

A previous study revealed the attentional demand of stair walking by measuring performance decrements in an auditory reaction time task while controlling gait cadence (Ojha et al. 2009). In the current study, however, participants did not receive explicit instruction on which task they should prioritize allowed them to use different strategies to find a solution to walk downstairs while dual tasking. Some participants chose to preserve locomotor behaviour and reduce the performance in the reaction time task, while others chose to preserve the performance in the reaction time task to the detriment of walking speed. The reduction in gait speed was associated with handrail use in order to increase safety on the stairs. Interestingly, individuals who employed a more cautious strategy exhibited this strategy whether or not the view of the stairs was facilitated. When participants chose to maintain walking speed, they tended to perform more gaze shifts downwards, which in some cases had an effect on the visual task performance. In other cases, participants seemed to find a balance point between the two tasks minimizing large performance decrements in both tasks. In real-world activities, this broad range of strategies is likely to happen and possibly related to individuals’ perception of threat in the task. Future studies investigating individual differences should further explore this issue and shed light into understanding challenges for balance control under dual-task conditions in everyday life activities as well as factors influencing locomotor strategies.

## Conclusions

In the presence of a central visual task, it appears that generally people do not look down as often when walking downstairs, supporting the capacity for use of peripheral visual information to guide stair walking. To deal with dual-task conditions, individuals adopt different strategies such as walking slower, using the handrails, and looking down. Walking on the midsteps of a staircase seems to require less from executive function, whereas visual attention may be required to detect the last transition via gaze shifts or overt visual attention through peripheral vision.
